# Asian house rats may facilitate their invasive success through suppressing brown rats in chronic interaction

**DOI:** 10.1186/s12983-017-0202-4

**Published:** 2017-04-12

**Authors:** Hong-Ling Guo, Hua-Jing Teng, Jin-Hua Zhang, Jian-Xu Zhang, Yao-Hua Zhang

**Affiliations:** 1 State Key Laboratory of Integrated Management of Pest Insects and Rodents in Agriculture, Institute of Zoology, Chinese Academy of Sciences, No.1-5 Beichen West Road, Chaoyang District, Beijing, 100101 China; 2 College of Life Sciences, University of Chinese Academy of Sciences, Beijing, 100049 China

**Keywords:** Closely related species, Invasive mechanism, Sexual attractiveness, Neuroendocrine molecules, Chronic stress

## Abstract

**Background:**

The Asian house rat (*Rattus tanezumi*) and the brown rat (*Rattus norvegicus*) are closely related species and are partially sympatric in southern China*.* Over the past 20 years, *R. tanezumi* has significantly expanded northward in China and partially replaced the native brown rat subspecies, *R. n. humiliatus*. Although invasive species are often more aggressive than native species, we did not observe interspecific physical aggression between *R. tanezumi* and *R. n. humiliatus*. Here, we focused on whether or not *R. tanezumi* was superior to *R. n. humiliatus* in terms of nonphysical competition, which is primarily mediated by chemical signals.

**Results:**

We performed two laboratory experiments to test different paradigms in domesticated *R. tanezumi* and *R. n. humiliatus*. In Experiment 1, we caged adult male rats of each species for 2 months in heterospecific or conspecific pairs, partitioned by perforated galvanized iron sheets, allowing exchange of chemical stimuli and ultrasonic vocalization. The sexual attractiveness of male urine odor showed a tendency (marginal significance) to increase in *R. tanezumi* caged with *R. n. humiliatus*, compared with those in conspecific pairs. Hippocampal glucocorticoid receptor (*GR*) and brain-derived nutrition factor (*BDNF*) mRNA were upregulated in *R. n. humiliatus* and *R. tanezumi*, respectively, when the rats were caged in heterospecific pairs. In Experiment 2, we kept juvenile male rats in individual cages in rooms with either the same or the different species for 2 months, allowing chemical interaction. The sexual attractiveness of male urine was significantly enhanced in *R. tanezumi*, but reduced in *R. n. humiliatus* by heterospecific cues and mRNA expression of hippocampal *GR* and *BDNF* were upregulated by heterospecific cues in *R. n. humiliatus* and *R. tanezumi*, respectively. Although not identical, the results from Experiments 1 and 2 were generally consistent.

**Conclusions:**

The results of both experiments indicate that nonphysical/chronic interspecific stimuli, particularly scent signals, between *R. n. humiliatus* and *R. tanezumi* may negatively affect *R. n. humiliatus* and positively affect *R. tanezumi*. We infer that chronic interspecific interactions may have contributed to the invasion of *R. tanezumi* into the range of *R. n. humiliatus* in natural habitats.

## Background

The brown rat (*Rattus norvegicus*), the Asian house rat (*R. tanezumi*), and the black rat (*R. rattus*) are three closely related commensal pests [1]. *R. norvegicus* has now spread from northern Asia to all continents except Antarctica, whereas *R. tanezumi* is mainly distributed in eastern, southern, and south-eastern Asia [1–3]. In China, *R. norvegicus* is widespread and has differentiated into four subspecies, including *R. n. norvegicus*, *R. n. soccer*, *R. n. humiliatus*, and *R. n. caraco*, whereas *R. tanezumi* typically lives south of the Yellow River and is sympatric with *R. n. norvegicus* and *R. n. soccer* [1, 4, 5]. *R. tanezumi* is also sympatric with *R. n. caraco* in the Korean peninsula [6]*. R. n. humiliatus*, which is the smallest of the four subspecies and lives mainly in Hebei Province in central North China, is the only subspecies geographically isolated from *R. tanezumi* [4, 7]; however, *R. tanezumi* has recently expanded its range to north of the Yellow River in the south of Hebei Province and partially replaced the native *R. norvegicus* subspecies (i.e., *R. n. humiliatus*) [8–10]. Several factors, including global warming and higher resistance to common rodenticides compared with brown rats, are believed to likely contribute to the invasive success of *R. tanezumi* [5, 11–15].

Exotic invasive species that successfully expand their range and displace native species appear to exhibit superiority in interspecific competition [16]. As closely related species are more likely to compete than those that are more distantly related, their competition, coexistence, and invasion have been extensively studied [16–20]. There are several types of interspecific competition, including indirect resource competition, interspecific aggression, interspecific territoriality, overgrowth, and chemical competition [16, 21]. In the case of rodents, invasive species are often more aggressive than native species [22]. While spreading from Asia to Europe and America during the Middle Ages, *R. norvegicus* generally displaced *R. rattus* in human settlements, where they out-competed *R. rattus* via physical interspecific interactions [11, 23]. It is logical that *R. norvegicus* is superior to *R. rattus* in terms of interspecific aggression, since the former is larger than the latter [1]; however, invasive *R. tanezumi* is generally smaller than native *R. n. humiliatus*. We did not observe that male *R. n. humiliatus* and *R. tanezumi* displayed interspecific aggression in dyadic encounters in a neutral arena (a common laboratory method to investigate aggressive behavior), and they can even live peacefully together for long periods of time when caged in interspecific male–male pairs [1, 19] (unpublished data). Thus, it is necessary to explore whether other types of interspecific competition (e.g., nonphysical interaction) contribute to the successful invasion of *R. tanezumi* into areas containing *R. n. humiliatus*.

Interspecific chemical interactions, including communication signaling and allelopathy, are widespread among prokaryotes, plants, and invertebrates, and are important in the invasion of exotic species [12, 14, 24]. For mammals, odor-mediated communication between different species (e.g., during predator and prey interactions) is also important, and the general laws of chemical ecology apply [25, 26]. Rodents use scent signals extensively in species recognition and interspecific competition [25, 27–30]. In rodents, scent signals, including urine volatile compounds and major urine proteins, are distinctive between closely related species and even between subspecies, including *R. n. humiliatus* and *R. tanezumi* [31–38] (unpublished data). The scent signals released by animals can function in heterospecific, as well as conspecific interactions, without the physical presence of the donor [37, 39–41]. Therefore, in rodents, where physical antagonism does not exist, interspecific competition may rely partially or completely on interspecific odor-based effects.

Interspecific competition can induce physiological stress responses and inhibitory effects on some phenotypic traits of competitors [20, 42]. In rodents, male scent signals have crucial roles in mediating sexual behavior and are often correlated with reproductive success [43–46]. If interspecific interactions induce strong chronic social stress, they can impair the attractiveness of the odor of male urine to females, and the production of urinary sex pheromones, and consequently disturb rodent reproductive behavior [47–50]. Conversely, changes in the sexual attractiveness of males subjected to interspecific competition may indicate stressful states in their competitors.

Competition-induced stress can activate the hypothalamic–pituitary–adrenal (HPA) axis to release endocrine hormones and neurotransmitters and alter gene expression in some regions of the rodent brain [25, 51–54]. After chronic competition, shifts in glucocorticoid levels are not always detectable in rodents [20]. The hippocampus is exquisitely sensitive to stressors, due to direct emotional input from the basolateral amygdala (BLA) and glucocorticoids (GCs), and because of its high density of GC receptors [55, 56]. The mRNA expression of hippocampal glucocorticoid receptor (*GR*), brain-derived neurotrophic factor (*BDNF*), and the BDNF receptor (*TrkB*) can be affected by stressors through modulation of the HPA axis and emotion-related input from the BLA [52–54]. Levels of *GR*, *BDNF*, and *TrkB* are closely related to emotional behavior, neuronal development, and plasticity, and can thus reflect emotional states in rodents [55, 57–59]. Stressors often impart different effects on adult and juvenile animals [60]. Early exposure to aversive stimuli often causes long-term alterations in many aspects of behavior, such as behavioral regulation, neuroendocrine responsiveness to stress, and mRNA expression of central nervous system genes related to behavioral change in rodents [61].

In the current study, we aimed to explore the potential roles of chronic nonphysical/chemical competition in the natural invasion process of *R. tanezumi* replacing *R. n. humiliatus* using laboratory experiments. Therefore, we performed two long-term experiments to test different paradigms in our laboratory. In Experiment 1, two adult male rats of the same or different species were caged together, partitioned by a perforated galvanized iron sheet; in Experiment 2, juvenile males of these two rat species were exposed to heterospecific or conspecific odors. To examine the effects of these exposures, we then evaluated changes in the sexual attractiveness of male urine odor and determined the mRNA levels of hippocampal *GR*, *BDNF*, and *TrkB*, as well as serum cortisol concentrations.

## Results

### Effects of chronic interspecific interaction on body weight

**Fig. 1:**
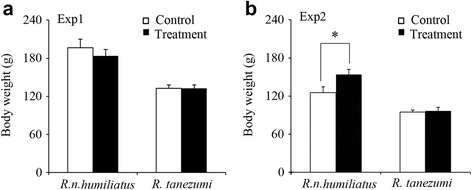
Comparison of final body weights (mean ± SE, g) between control and treatment groups of *R. norvegicus humiliatus* or *R. tanezumi* in Experiment 1 (Exp1) (**a**) (*n*_control_ = *n*_treatment_ = 9 for *R. n. humiliatus* and *n*_control_ = 9, *n*_treatment_ = 10 for *R. tanezumi*.) and Experiment 2 (Exp2) (**b**) (*n*_control_ = *n*_treatment_ = 6 for *R. n. humiliatus*; *n*_control_ = 7 and *n*_treatment_ = 8 for *R. tanezumi*). Independent samples *t*-test or Mann–Whitney *U*-test; *p ≤ 0.05

Experiment 1: After two months of interaction, the body weight was not significantly different between the control and treatment groups of either *R. n. humiliatus* or *R. tanezumi* (Fig. 1a).

Experiment 2: After two months of interaction, the body weight of immature males exposed to a heterospecific odor was higher than that of the control group (*t* = 2.232, *n* = 6 for each group, *p* = 0.050) in *R. n. humiliatus*, whereas body weight did not differ between the control and treatment groups in *R. tanezumi* (Fig. 1b).

### Sexual attractiveness of male urine odor

**Fig. 2:**
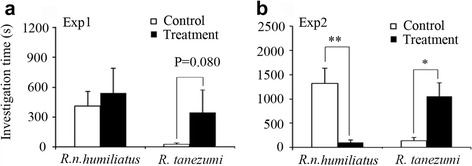
Investigation time (mean ± SE, sec) spent by female *R. norvegicus humiliatus* or *R. tanezumi* on conspecific male urine samples between control and treatment groups in Experiment 1 (Exp1) (**a**) (*n* = 15 for test female rats of each species), and Experiment 2 (Exp2) (**b**) (*n* = 18 for test females of each species). Paired *t*-test or Wilcoxon signed-rank test; *p < 0.05, **p < 0.01

Experiment 1: In *R. n. humiliatus*, female attraction to male urine did not differ between the control and treatment groups (Fig. 2a), while in *R. tanezumi*, females exhibited a trend towards preferring males caged with *R. n. humiliatus* over those caged with their own species (*z* = 1.726, *n* = 12, *p* = 0.080, marginal significance) (Fig. 2a).

Experiment 2: In *R. n. humiliatus*, heterospecific odor stimulation significantly suppressed the sexual attractiveness of male urine to conspecific females compared with conspecific odor stimulation (*z* = 2.884, *n* = 16, *p* = 0.004) (Fig. 2b). Conversely, the sexual attractiveness of male urine was significantly greater after heterospecific odor stimulation in *R. tanezumi* (*z* = 2.373, *n* = 18, *p* = 0.018) (Fig. 2b).

### Serum cortisol and testosterone levels

**Fig. 3:**
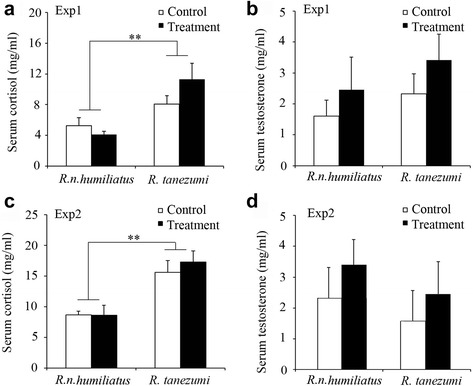
Serum cortisol and testosterone concentration (mean ± SE) in *R. norvegicus humiliatus* or *R. tanezumi* in Experiment 1 (Exp1) (*n*_control_ = 12, *n*_treatment_ = 10 for *R. n. humiliatus*; *n*_control_ = 12 and *n*_treatment_ = 9 for *R. tanezumi* (**a** and **b**) and Experiment 2 (Exp2) (*n*_control =_ *n*_treatment_ = 6 for *R. n. humiliatus*; *n*_control_ = 7 and *n*_treatment_ = 8 for *R. tanezumi*) (**c** and **d**). Independent samples *t*-test or Mann-Whitney *U*-test; *p < 0.05, **p < 0.01

Experiment 1: A radioimmunoassay demonstrated that serum testosterone and cortisol levels were not significantly different between the control and treatment groups for either *R. n. humiliatus* or *R. tanezumi* (3a and b). However, serum cortisol concentration was higher in both the control and treatment groups of *R. tanezumi* than those of *R. n. humiliatus* (control group: *t* = 2.938, *n* = 12 for each species, *p* = 0.008; treatment group: *t* = 3.395, *n* = 9-10 for each species, *p* = 0.004) (Fig. 3a)*.*

Experiment 2: Similarly, interspecific odor stimulation had no apparent effect on serum testosterone or cortisol levels within species for either *R. n. humiliatus* or *R. tanezumi* (Fig. 3c and d)*. R. tanezumi* also had a higher serum cortisol concentration than *R. n. humiliatus* in both the control (*t* = 3.173, *n* = 6-7 for each species, *p* = 0.009) and treatment (*t* = 3.445, *n* = 6-8 for each species, *p* = 0.005) groups (Fig. 3c)*.*

### Gene expression of *GR* and *BDNF* in the hippocampus

**Fig. 4:**
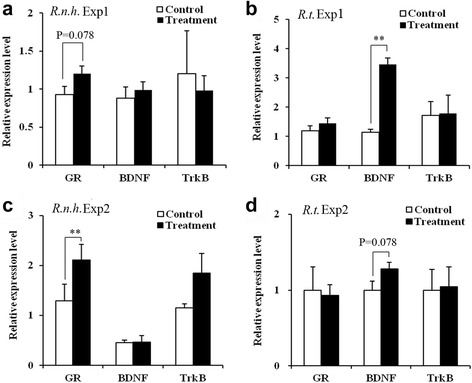
Comparison of hippocampal expression of *GR*, *BDNF*, and *TrkB* mRNA (mean ± SE) between control and treatment groups of *R. norvegicus humiliatus* (*R.n.h.*) (**a** and **c**) or *R. tanezumi* (*R.t.*) (**b** and **d**) in Experiment 1 (Exp1) (*n*_control_ = *n*_treatment_ = 8 for *R.n.h.* and *n*_control_ = *n*_treatment_ = 8 for *R.t.*) and Experiment 2 (Exp2) (*n*_control =_ *n*_treatment_ = 6 for *R.n.* *h.*; *n*_control_ = 7 and *n*_treatment_ = 8 for *R.t.*). Independent samples *t*-test or Mann-Whitney *U*-test; *p < 0.05, **p < 0.01

Experiment 1: Quantitative real-time PCR demonstrated that *GR* mRNA expression was slightly upregulated in the hippocampus of *R. n. humiliatus* rats exposed to heterospecific stimuli (*t* = 1.901, *n* = 8 for each group, *p* = 0.078, marginal significance), whereas hippocampal *BDNF* was significantly upregulated in *R. tanezumi* exposed to heterospecific stimuli (*z* = 3.361, *n* = 8 for each group, *p* = 0.001) (Fig. 4a and b). Hippocampal *TrkB* mRNA expression exhibited no significant changes within either rat species (Fig. 4a and b).

Experiment 2: Similar to the results of Experiment 1, hippocampal *GR* mRNA expression (*t* = 4.125, *n* = 6 for each group, *p* = 0.009) in *R. n. humiliatus* and *BDNF* mRNA expression (*t* = 1.911, *n* = 7-8 for each group, *p* = 0.078, marginal significance) in *R. tanezumi* were upregulated (Fig. 4c and d). Hippocampal *TrkB* mRNA expression showed no change within either rat species.

## Discussion

Our results from binary choice tests suggest that chronic nonphysical competition between *R. tanezumi* and *R. n. humiliatus* exerts completely opposite effects on their male scent signals, which were enhanced in *R. tanezumi*, but inhibited in *R. n. humiliatus*. The results from Experiments 1 and 2 were not identical; however, they were generally consistent with one another. As sexual attractiveness is often correlated with reproductive success and fitness in male animals, the reproductive success of *R. n. humiliatus* may be suppressed due to reduced sexual attractiveness, and augmented in *R. tanezumi* due to increased sexual attractiveness resulting from chronic interspecific competition. Such asymmetric competition effects on scent signals and reproductive behavior may have facilitated the invasive *R. tanezumi* population to replace that of the native *R. n. humiliatus* [41, 43, 62–65]. Our results, therefore, warrant further investigation in the laboratory and the field [46, 66]. Although rodents can emit species-specific ultrasonic vocalizations that are behaviorally important for mating, nursing, aggression, defense, and emotion within species, the effects of ultrasound-mediated chronic competition between closely related species appear to be very weak [67–69]. Therefore, we believe that nonphysical chronic interspecific interactions between *R. tanezumi* and *R. n. humiliatus* are likely to have been primarily mediated by chemical signals in our experiments.

In addition, chronic stress often impairs the sexual attractiveness of urine odor and decreases the levels of volatile pheromones in male mouse urine [47–49]. For example, both the presence of a predator and its scent can inhibit the sexual attractiveness of male mouse urine [48]. However, low predation risk, reflected as a low dose of predator scent, has a positive effect, boosting the sexual attractiveness of male mouse urine [36, 47, 70]. In intraspecific male–male competition, two opponents can form a stable dominance–submission relationship, in which the dominant partner has more volatile pheromones in the urine and greater sexual attractiveness compared with the submissive partner [49, 71]. Here, particularly in Experiment 2, the sexual attractiveness of male urine was augmented in *R. tanezumi*, but suppressed in *R. n. humiliatus*, as a result of exposure to heterospecific stimuli, indicating that *R. n. humiliatus* may be stressed by *R. tanezumi*. Coincidentally, juvenile *R. n. humiliatus* rats exposed to *R. tanezumi* odor gained more body weight than those exposed to their own species in Experiment 2, possibly reflecting the influence of a stressor [44, 72].

The influence of nonphysical competition-induced stress was further confirmed by the observation of upregulated hippocampal *GR* mRNA levels in *R. n. humiliatus* in response to *R. n. humiliatus* cues. As reported in previous studies of competition stress, we detected alterations in the expression of *GR* and *BDNF* mRNA in the hippocampus, but no differences in blood cortisol and testosterone levels in *R. n. humiliatus* experiencing chronic stress due to the presence of *R. tanezumi* [20, 55, 56]. *GRs* are key mediators of the neuroendocrine response to stress, and stressor-specific alterations in *GR* mRNA levels are more pronounced in male than in female rodents [73]. Hippocampal *GR* has been implicated in negative feedback inhibition of the HPA axis and can mediate the deleterious effects of blood glucocorticoids on hippocampal neuron survival and function [52, 59]. The expression of hippocampal *GR* can be affected by stressful stimuli and is adaptively downregulated to protect the hippocampus from glucocorticoid hypersecretion in acute stress and in the early stages of chronic stress [52, 59]. In the late stages of chronic stress, blood glucocorticoid may return to control levels and hippocampal *GR* expression may be upregulated due to the buffering effect of repeated stress and habituation effects [74]. Therefore, the upregulation of hippocampal *GR* mRNA observed in *R. n. humiliatus* in our study may indicate that this species was physiologically impaired by long-term interaction with *R. tanezumi.*

In contrast, hippocampal *BDNF* mRNA levels were upregulated in *R. tanezumi*, particularly in Experiment 1, after exposure to *R. n. humiliatus*. The neurotrophic factor, *BDNF*, is associated with neuronal development, survival, and plasticity, and has been found to decrease in response to acute and mild chronic stress, which may contribute to the neuronal atrophy/death observed in rodents suffering from chronic stress [57, 58]. Conversely, an enriched environment (EE) can ameliorate stress-induced symptoms, such as anxious behavior and increase hippocampal neurogenesis and *BDNF* protein levels in mice and rats [36, 70]. In this study, heterospecific cues from *R. n. humiliatus* enhanced hippocampal *BDNF* gene expression in *R. tanezumi*, and an EE may therefore improve cognitive function, spatial memory, and local behavioral adaptation of invasive *R. tanezumi*. For example, it is conceivable that, if immature *R. tanezumi* rats of pioneer populations disperse into the range of *R. n. humiliatus*, the resulting heterospecific cues will promote the development and survival of *R. tanezumi*. Moreover, repeated encounters between adult rats of these two species during the invasion process may improve the neuroendocrine state and adaptive behavior of *R. tanezumi*.

## Conclusions

The results of both experiments conducted in this study imply that chronic nonphysical interspecific stimuli, particularly scent signals, can have asymmetric effects on *R. n. humiliatus* and *R. tanezumi*, leading to detrimental effects on the sexual attractiveness and neuroendocrine system of the former, and favorable effects on the same factors in the latter. Thus, we infer that chronic interspecific interactions may contribute to the invasive success and northward expansion of *R. tanezumi* and the decline of native *R. n. humiliatus* populations in natural habitats. These results warrant further investigation in the field.

## Methods

### Animals

Wild *R. tanezumi* and *R. n. humiliatus* were captured from Shanxi Province and Beijing (China), respectively. Each rat species was maintained as an outbred colony of 300–400 rats in our laboratory. The rats used were of the third generations, weaned at 4 weeks of age, and caged in groups of same sex siblings prior to use. All animals were kept in plastic rat cages (37 × 26 × 17 cm), in two separate rooms (14:10 h light: dark photoperiod, lights on at 5:00 am) and were maintained at 25 °C ± 2 °C. Food (standard rat chow) and water were provided *ad libitum*.

#### Experiment 1 (assessment of chemical signals and ultrasonic vocalization stimuli in adult animals)

**Fig. 5:**
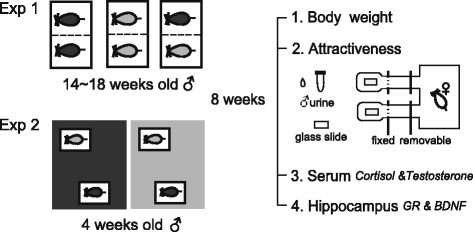
Experimental set up. In Experiment 1, from 22 adult male rats of each rat species aged from 14 to 18 weeks, 10 were paired with the other species and the others were paired with their own species as a control. All pairs of rats were housed in the same cage for 2 months, and partitioned by perforated galvanized iron sheets. In Experiment 2, one group (*n* = 8) was kept in a room with the other species, and the other group (*n* = 7) remained in a room with its own species as a control. Each rat room was 15 m^2^ and contained approximately 120 rats of the same species. Eight weeks later, body weight, attractiveness of urine, serum cortisol and testosterone, and hippocampal *GR* and *BDNF* expression were measured. The attractiveness of male urine was determined using a two-choice box, consisting of a plastic (start box) and two Plexiglas choice tubes (internal diameter, 7.5 cm; length, 50 cm). The choice tubes were symmetrically connected to the start box and each tube had a removable perforated galvanized iron sheet partition 5 cm away from the box to control rat access. An odorant presentation compartment partitioned by a perforated galvanized iron sheet from the other part of the tube was at the distal end of each 10 cm tube. We painted the male urine on a glass slide, and then placed the slide in the presentation compartment

Twenty-two adult male rats of each rat species were randomly selected from the colonies at 14–18 weeks of age, and 10 paired with the other species and the others paired with their own species as a control. All pairs of rats were housed in the same cage for 2 months, and all cages were partitioned with perforated galvanized iron sheets containing one 0.3 cm diameter hole per cm^2^, to allow chemical and ultrasonic interactions (Fig. 5). Body weights were not significantly different between control and treatment groups of the same species (*R. n. humiliatus*: 169.4 ± 9.069 g vs. 172.5 ± 8.303 g, *t* = 0.014, *p* = 0.989; *R. tanezumi*: 135.4 ± 7.248 g vs. 127.2 ± 4.397 g, *t* = 1.281, *p* = 0.218).

The sexual attractiveness of the odor of the urine from heterospecific or conspecific caged males was assessed using twelve estrous female rats of each species. All female subjects were between 14 and 18 weeks of age and had estrous cycles of 4–5 days, as determined by a vaginal smear examination. Females were used in experiments on the days that they came into estrus. Sixteen female rats of each species were used as urine recipients in Experiment 1.

#### Experiment 2 (assessment of the effects of odor stimulus on young animals)

Fifteen immature males (4 weeks old) of each species were individually caged and randomly assigned into two groups for 2 months. One group (*n* = 8) was kept in a room with the other species, while the other group (*n* = 7) remained in a room with its own species, as a control. Each rat room was 15 m^2^ and contained approximately 120 rats (Fig. 5). Body weights were not significantly different between the two groups of the same species (*R. n. humiliatus*: 50.66 ± 3.972 g vs. 49.31 ± 4.533 g, *t* = 0.225, *p* = 0.826; *R. tanezumi*: 47.36 ± 6.177 g vs. 46.03 ± 9.010 g, *t* = 0.338, *p* = 0.740). Eighteen female rats of each species in estrus were used as urine recipients in Experiment 2.

### Urine collection

Within 3 days after the chronic interspecific interaction experiments, we individually collected rat urine using clean metabolic rat cages during the dark phase of the light cycle. Urine collection continued for 8 h daily. The urine from the metabolic cage was collected in a tube immersed in an ice box. Standard rat chow and water were freely available. Urine samples were stored at -20 °C until use. Metabolic cages were washed thoroughly with water between urine collections.

### Behavioral tests of sexual attractiveness

Olfactory preference tests were conducted in a two-choice box that consisted of a plastic rat cage that served as a start box and two Plexiglas choice tubes (internal diameter, 7.5 cm; length, 50 cm). The two choice tubes were symmetrically connected to the long side of the start box and each tube had a removable perforated galvanized iron sheet partition 5 cm away from the box to control rat access. An odorant presentation compartment partitioned by a perforated galvanized iron sheet from the other part of the tube was at the distal end of each 10 cm tube (Fig. 5).

Female rats were first test acclimated in the start box for 30 min, then a microscope slide with a urine sample (20 μL) was placed into the odorant presentation compartment of each tube. Female rats were simultaneously exposed to two urine samples from males conspecific to them, where one of the samples was from a male that had been housed with a conspecific male, and the other with a heterospecific male. We immediately opened the door to allow the females to freely respond to the urine. Between trials, the start box and choice tubes were cleaned thoroughly with water and 75% ethanol. All tests were recorded on video. Investigation times (i.e., the time each female spent in a choice tube) were determined from video replay using a Noldus ethovision XT system (Noldus, Wageningen, The Netherlands). We recorded the investigation time for 30 min in Experiment 1 and 1 h in Experiment 2 after the female initially entered either of the choice arms. If a test female did not enter either choice tube within 30 min (i.e., investigation time = 0), we did not use the data.

### Blood and tissue sampling

Two days after urine collection, all rats were decapitated (within 3 min) and blood samples immediately collected. The hippocampus was immediately dissected, rapidly frozen in liquid nitrogen, and stored at -80 °C until use. Blood samples were incubated at 4 °C in a refrigerator for 12 h and then centrifuged at 3000 rpm for 15 min for serum collection. Serum samples were stored at -80 °C until use. In Experiment 1, individual hippocampi from eight males were used for RNA isolation and quantitative real-time PCR; in Experiment 2, all of the hippocampi were used.

### Cortisol and testosterone analysis

Serum samples were analyzed in duplicate for cortisol using an Iodine[^125^I] cortisol RIA kit and for testosterone using an Iodine[^125^I] Testosterone RIA Kit (Beijing North Institute of Biological Technology, China). In detail, 100 μL of iodine-125 labeled cortisol (or testosterone) was incubated with 50 μL of serum at 37 °C for 1 h in a water bath. Then, 500 μL of immune separating agent was added to each sample tube and samples incubated at room temperature for 15 min. Sample tubes were then centrifuged at 3800 rpm for 15 min and the supernatant discarded. The remaining radioactivity bound to the tube was measured using a gamma scintillation counter calibrated for iodine-125 using a radioimmunoassay system (XH6080, Xi'an Nuclear Instrument Factory, Xi'an, China). For both cortisol and testosterone, the intra-assay coefficients of variation were less than 10%, and the inter-assay coefficients of variation were less than 15%.

### Quantitative real-time PCR

Isolation of total hippocampal RNA was performed using Trizol reagent (Invitrogen, Life Technologies, Grand Island, NY, USA) according to the manufacturer’s instructions. Total RNA concentration was determined using a NanoDrop spectrophotometer (Thermo Fisher Scientific Inc., Waltham, USA). Reverse-transcription was performed using a PrimeScript® RT reagent Kit With gDNA Eraser (Perfect Real Time) (Takara Bio Inc., Dalian, China), following the manufacturer’s protocol. The resulting cDNA was amplified using an Mx3005P quantitative PCR system (Stratagene, La Jolla, CA, USA) and the relative abundance of the mRNA of the target genes determined using a SYBR Green RealMasterMix Kit (Tiangen, Beijing, China) according to the manufacturer’s instructions. PCR primers were designed using NCBI Primer Blast (http://www.ncbi.nlm.nih.gov/tools/primer-blast) and the sequences are listed in Table 1. The housekeeping gene, *GAPDH*, was used as a control to normalize the relative mRNA levels. Data were analyzed as previously described [35].

**Table 1:** Primer sequences for real-time PCR

Gene	Forward Primer 5′–3′	Reverse Primer 5′–3′
*GAPDH*	GACAATGAATATGGCTACAGCAAC	TTTATTGATGGTATTCGAGAGAAGG
*GR*	AGGCAGTGTGAAATTGTATCCCAC	GAGGCTTACAATCCTCATTCGTGT
*BDNF*	GAAGGGCCAGGTCGATTAGGTG	GACGGAAACAGAACGAACAGAA
*TrkB*	GAGACGAAATCCAGCCCCGACAC	CACAGACTTCCCTTCCTCCACCG

### Statistical analyses

The distributions of raw data were examined using Kolmogorov–Smirnov tests. If data were normally distributed, *t*-tests for paired-samples were used for the behavioral data and independent *t*-tests were used for the body weight, serum hormone and mRNA expression data. If the data were not normally distributed, Wilcoxon signed-rank and Mann–Whitney *U* tests were used. All statistical analyses were conducted using the SPSS software package (v15.0, SPSS Inc., Chicago, IL, USA). Alpha was set at *P* ≤ 0.05.
